# Robot-assisted versus traditional surgery in the treatment of intertrochanteric fractures: a meta-analysis

**DOI:** 10.1007/s11701-024-01979-7

**Published:** 2024-05-23

**Authors:** Jiaxiao Shi, Jiaxin Shen, Chaochao Zhang, Wei Guo, Fangfang Wang

**Affiliations:** 1 Department of Orthopaedics, Hebei Province Cangzhou Hospital of Integrated Traditional Chinese Medicine-Western Medicine, Cangzhou, China; 2 Hebei Key Laboratory of Integrated Traditional and Western Medicine in Osteoarthrosis Research (Preparing), Cangzhou, China; 3 Department of Intensive Care Unit, Cangzhou Central Hospital, Cangzhou, 061001 China

**Keywords:** Robot-assisted, Intertrochanteric fractures: a meta-analysis

## Abstract

Intramedullary nail fixation of intertrochanteric fractures assisted by orthopedic surgical robot navigation is a new surgical method, but there are few studies comparing its efficacy with traditional intramedullary nail fixation. We aimed to assess whether robot-assisted internal fixation confers certain surgical advantages through a literature review. PubMed, EMBASE, Cochrane Library, China National Knowledge Infrastructure (CNKI) and Wan fang Data Knowledge service Platform were searched to collect randomized and non-randomized studies on patients with calcaneal fractures. Five studies were identified to compare the clinical indexes. For the clinical indexes, the technology of robot-assisted is generally feasible, in time to operation, intraoperative fluoroscopy times, blood loss, pine insertion, tip apex distance (TAD), and Harris score (*P* < 0.05). However, on the complication and excellent and good rate after operation did not show good efficacy compared with the traditional group (*P* > 0.05). Based on the current evidence, For the short-term clinical index, the advantages of robot-assisted are clear. The long-term clinical effects of the two methods are also good, but the robot-assisted shows better. However, the quality of some studies is low, and more high-quality randomized controlled trials (RCTs) are needed for further verification.

## Introduction

Hip fracture is one of the most common fractures in the elderly. With the aging of the population, the incidence of hip fractures is increasing year by year, and 41–50% of these fractures are intertrochanteric fractures in the elderly [1, 2]. It limits the patient’s mobility, leading to chronic pain and disability and reduced quality of life [3]. The treatment of intertrochanteric fractures is mainly divided into conservative treatment and surgical treatment [4]. Because most of the patients are elderly, the incidence of complications is high, so most doctors think that surgical treatment is needed [5, 6]. Proximal femoral intramedullary nail is one of the most commonly used surgical options for the fixation of femoral intertrochanteric fractures [7]. Through surgical fixation, bed-function training can be carried out early, and the occurrence of various complications caused by long-term bed rest can be reduced. How to improve the accuracy of the guide pin, how to reduce the surgical trauma of patients, and how to reduce the X-ray exposure time of both doctors and patients are important. With the improvement of medical technology and the rapid development of minimally invasive surgery, surgical robots have been gradually used in clinical treatment of various departments because of their good stability, operation flexibility, and accurate movement [8, 9]. Orthopedic surgical robots are mainly used in the spine and joint direction [10, 11]. Through the intraoperative X-ray and CT-image acquisition, the surgical site is matched with the robot operation position, so as to guide the doctor to perform intraoperative positioning and internal fixation placement, shorten the operation time and reduce the surgical trauma. To analyze the advantages and disadvantages of intramedullary nailing assisted by orthopedic surgical robot navigation in the treatment of intertrochanteric fractures. Many related experiments are testing and verifying this issue. Therefore, we collected relevant articles and performed a meta-analysis to provide a basis for doctors’ decisions [12–16].

## Materials and methods

### Search strategy

Two investigators reviewed literature based on the patient/ population intervention comparison outcome model principles. The keywords used in these searches were “robot” and “intertrochanteric fracture” which were searched in “All fields” in the PubMed, EMBASE, The Cochrane Library, CNKI (China National Knowledge Infrastructure) and Wan fang Data Knowledge Service Platform, and these terms were connected by using the search term “AND” and “OR”. The initial time periods and languages of the searches were not limited, they just ruled the deadline to January15, 2024. Finally, the eligible articles were chosen by the selection criteria. For disagreements studies, we submitted them to more experienced individuals to decide on the selection.

### Selection criteria

- **(1)** Participants: intertrochanteric fracture was diagnosed by imaging and specialist physical examination; surgical treatment was performed after obtaining the informed consent of the patients and their families. Pathologic fracture; severe hemodynamic instability requiring emergency surgical intervention; combined with other organ injury or fracture; patients with general conditions or concomitant diseases that are difficult to tolerate surgery.
- **(2)** Intervention and comparison: The group where patients received robot-assisted fixation is intervention group, and patients who just accept conventional surgery were filed in control group. Patients who had received others operation forms were excluded.
- **(3)** Outcomes: The clinical indexes involving the time to operation, intraoperative fluoroscopy times and blood loss. The guide pine insertion, tip apex distance (TAD), complication, excellent and good rate and Harris score after surgery were used as the research outcomes of the study.
- **(4)** Study design: Randomized controlled trials and retrospective study that compared the robot-assisted fixation with traditional fixation was considered qualified.

### Quality assessment

We used the Cochrane Handbook to evaluated the risk of randomized controlled trails data, and according to the result we classified the studies to the three levels. In additional, for the retrospective study, we used the Newcastle-Ottawa Scale (NOS) to evaluated the quality of articles. We consider studies with NOS scores greater than 5 to indicate moderate to high-quality studies.

### Data extraction

We extracted the data on first author, publication time, country, study design, number of patients, mean of age, the percentage of sex, follow-up examination(month) and Interventions. When the disagreement occurred the third reviewer give the final decision.

### Data analysis and statistical methods

We used the Review Manager, version 5.3 to analysis the data. The odds ratio (OR) was used to calculated the dichotomous outcomes. Moreover, we use *I*^2^ values to assess heterogeneity between articles. If *I*^2^ > 50, a random effect model is used, otherwise a fixed effect model is selected.

## Results

### Search results

**Fig. 1:**
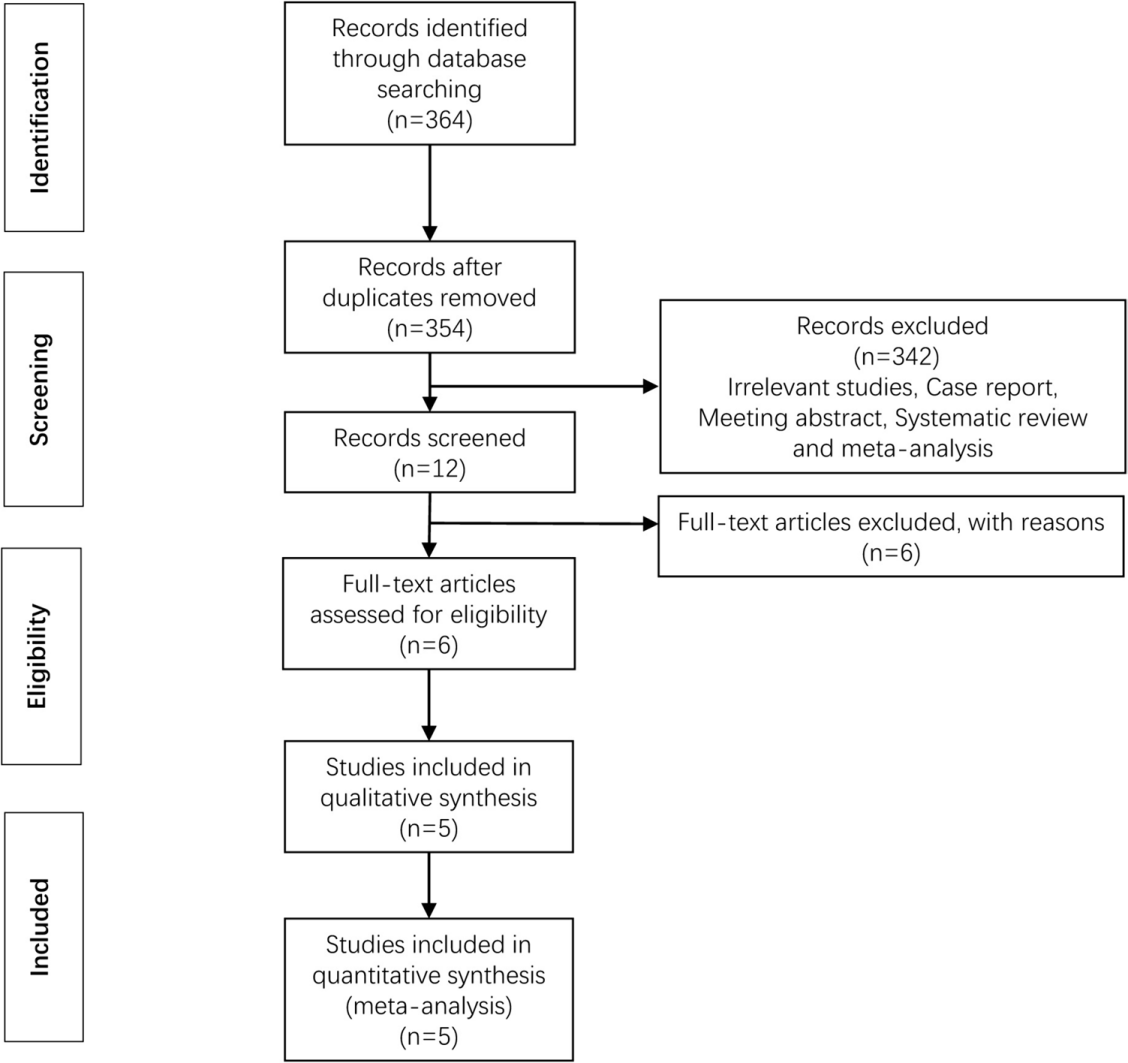
Flowchart of the study selection process

A total of 364 articles were identified by our query method, of which 60 were from the PubMed, 6 from Embase,1 from the Cochrane Library, 294 from CNKI and 3 from Wan Fang Data Knowledge Service Platform. In these articles, 10 studies were eliminated because of duplicate checking. Investigators picked out 342 articles according to the meaning of the title and abstract. Finally, we adopted five articles through reading and understanding the full text. The whole document screening process was reflected in Fig. 1.

### Risk of bias assessment

**Fig. 2:**
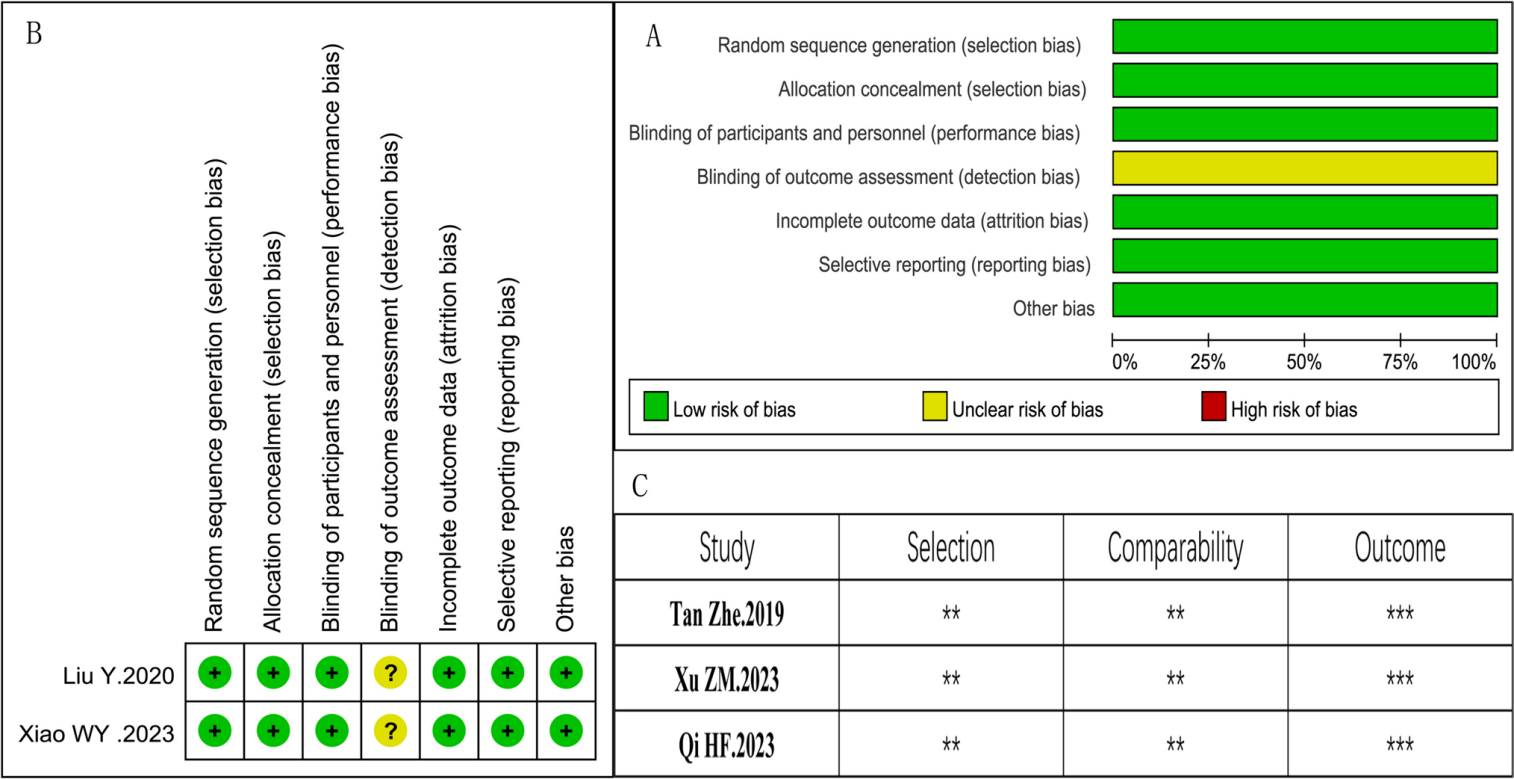
Risk of bias assessment of RCT studies. **A** Risk of bias graph. **B** Risk of bias summary; Quality assessment score of the retrospective study **C**

In these incorporated documents, the methodological quality of two types of experiments were evaluated according to their respective evaluation criteria which were mentioned in the methods and materials. Among the included studies, except two is RCT, the rest are retrospective studies. The RCT are considered better quality. The retrospective studies were considered as good quality, the detail contents were exhibited in Fig. 2.

### Study characteristics

Of the five articles included, two were RCTs, three of which were retrospective study, and these were single-center studies. Almost every article provided general characteristics of the study population. Each article provided that the study population was all as patients. Finally, our study will include 352 people. Among them, 131 patients used traditional group, 221 patients used robot-assisted group. The characteristics of these documents will be presented in Table. 1.

**Table 1:** Summary of characteristics in the studies included

Author/year	Tan Zhe.2019	Xiao WY .2023	Liu Y 2020	Xu ZM 2023	Qi HF 2023
Country	China	China	China	China	China
Study design	Retro	RCT	RCT	Retro	Retro
No. of patients (RA/traditional)	28/31	25/25	10/10	31/29	36/36
Age (Y) (RA/traditional)	77.32 ± 6.75/76.58 ± 6.66	71.6 ± 9.5/72.4 ± 11.0	82.3 ± 4.3/85.2 ± 3.2	78.7 ± 9.3/79.0 ± 7.3	77.58 ± 9.28/80.31 ± 9.13
Male (No.) (RA/traditional)	13/17	9/13	4/3	11/12	16/18
Follow-up examination(M) Interventions	12	12	12	6	12
	RA/traditional	RA/traditional	RA/traditional	RA/traditional	RA/traditional

*RA* robot assisted, *RCT* randomized controlled trial, *Retro*: retrospective study, *Nr* Not reported, *D* Day, *M* Month, *Y* Year, *No.* number

### Outcomes of meta-analysis

#### Operation time

**Fig. 3:**
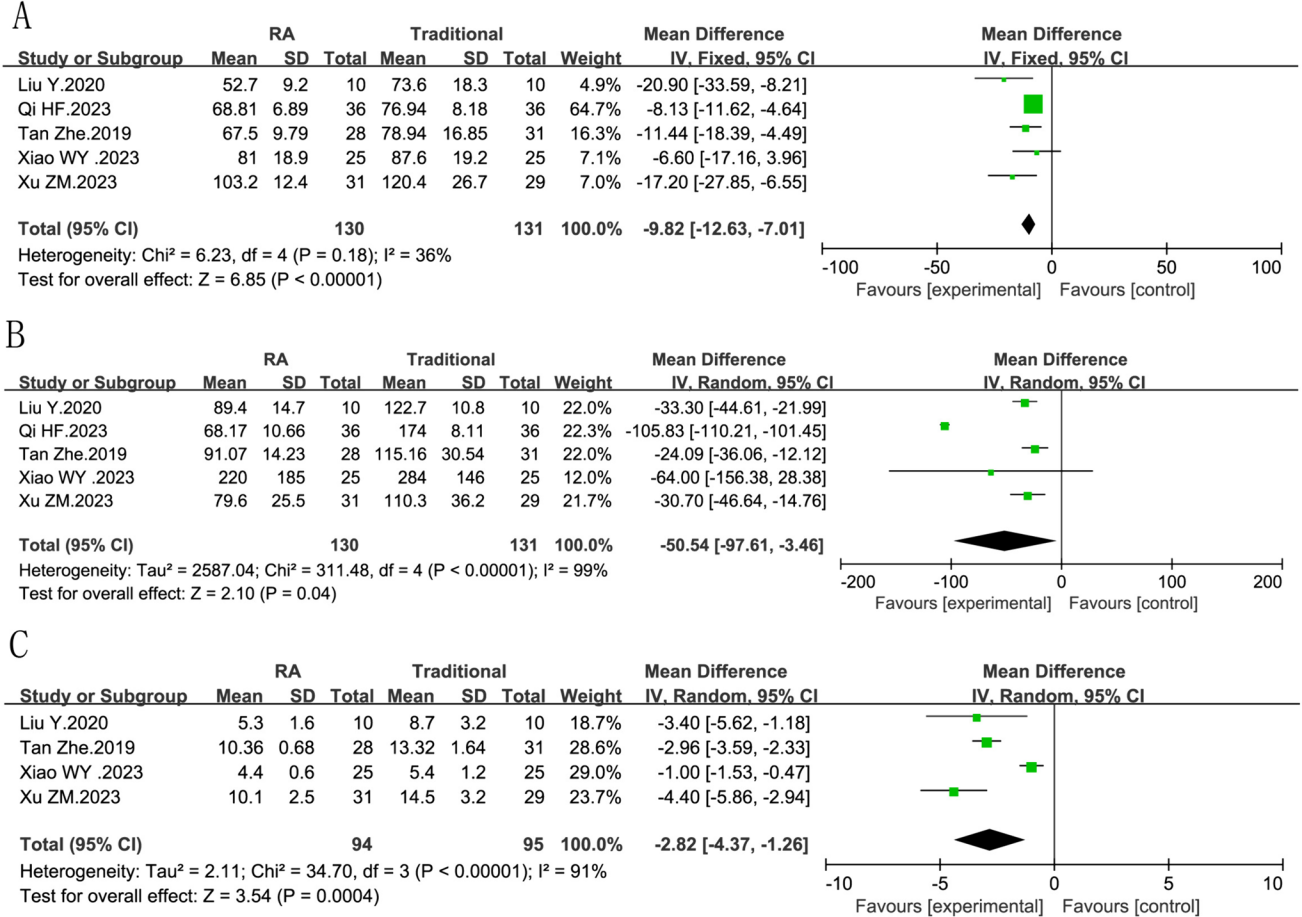
Forest plot diagram showing the operative time, intraoperative blood loss and intraoperative fluoroscopy times. **A** Operative time of robot-assisted vs traditional fixation. **B** Intraoperative blood loss of robot-assisted vs traditional fixation. **C** intraoperative fluoroscopy times of robot-assisted vs traditional fixation

Three reports provided data (*n *= 261) on operation time. The fixed effect model was used, and slight significant heterogeneity was found (*I*^2^ = 36%, *P *= 0.18). The experimental group took less time than the control group (RR = −9.82, 95% CI −12.63, −7.01, *P*<0.00001). (Fig. 3A)

#### Intraoperative blood loss

There are five reports offering data (*n *= 261) on intraoperative blood lose. A random effect model was used, and high heterogeneity was found (*I*^2^ = 99%, *P *< 0.00001). Therefore, robot-assisted fixation has advantages over the traditional group in terms of blood lose. (RR = −50.54, 95% CI −97.61, −3.46, *P *= 0.04). (Fig. 3B)

#### Intraoperative fluoroscopy times

There are four reports offering data (*n *= 189) on intraoperative fluoroscopy times. A random model was used, and high heterogeneity was found (*I*^2^ = 91%, *P *<0.00001). Therefore, robot-assisted fixation has obvious advantages over the traditional in terms of intraoperative fluoroscopy times. (RR = −2.82, 95% CI −4.37, −1.26, *P *= 0.0004). (Fig. 3C)

#### Pine insertion

**Fig. 4:**
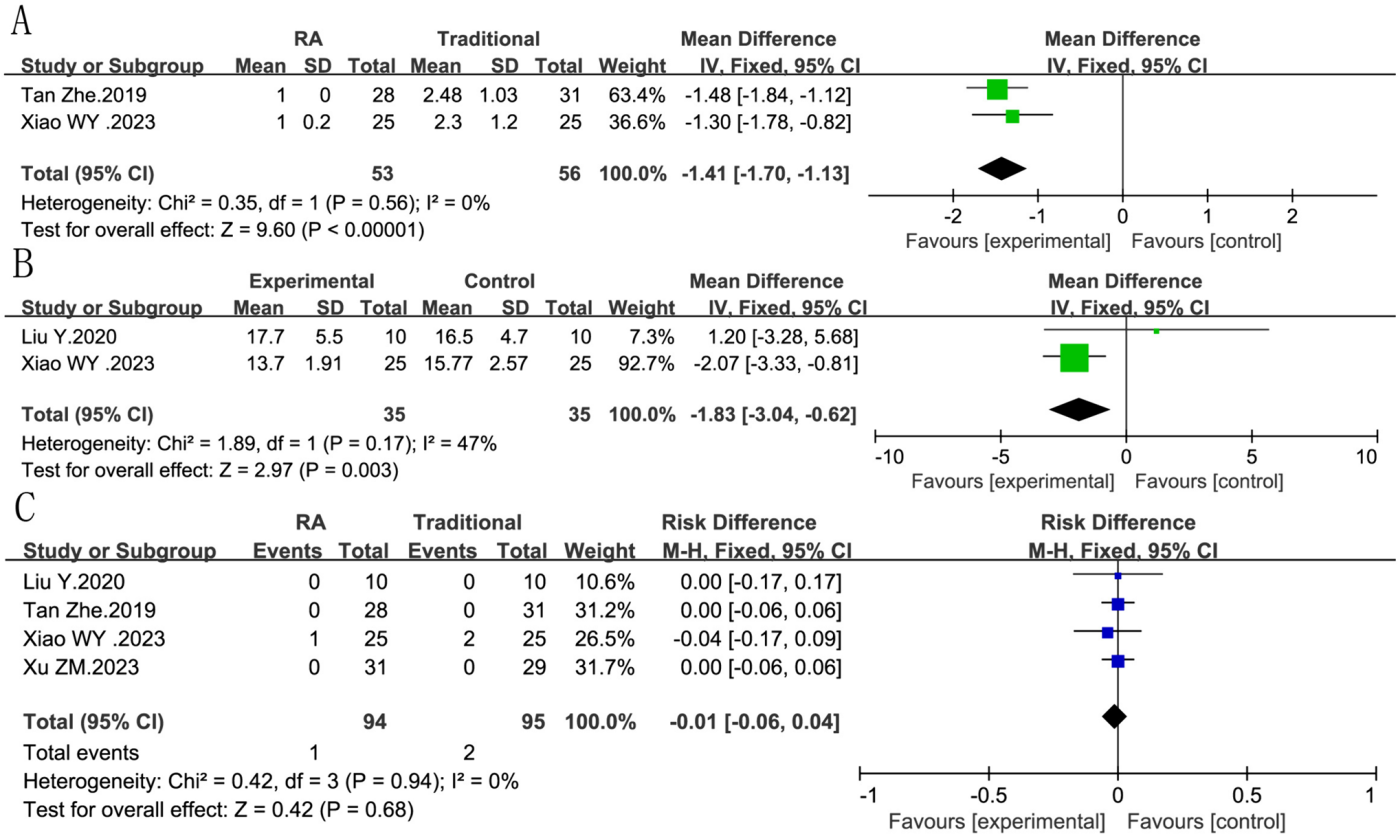
Forest plot diagram showing the pine insertion, tip apex distance (TAD), and complication. **A** Pine insertion of robot-assisted vs traditional fixation, **B** ip apex distance, **C** complication of robot-assisted vs traditional fixation

Two articles report relevant data applied to pine insertion. We still used fixed model, and had no heterogeneity was found (*I*^2^ = 0%, *P *= 0.56). The advantage of the experimental group was shown (RR = −1.41, 95% CI −1.70, −1.13, *P *<0.00001) (Fig. 4A).

#### Tip apex distance (TAD)

Two studies with 70 patients supported the indexes of TAD. A fixed model was used, and a moderate heterogeneity was appeared (*I*^2^ = 47%, *P *= 0.17). The accuracy of screw placement in the robot-assisted group was higher than that in the traditional group (RR = −1.83, 95% CI −3.04, −0.62, *P *= 0.003). (Fig.4B)

#### Complication

Four articles report relevant data applied to complication. We still used a fixed effect model, and had no heterogeneity was found (*I*^2^ = 0%, *P *= 0.94). The two groups showed the same results (RR = −0.01, 95% CI −0.06, 0.04 *P *= 0.68). (Fig. 4C)

#### Hip function at 1 year

**Fig. 5:**
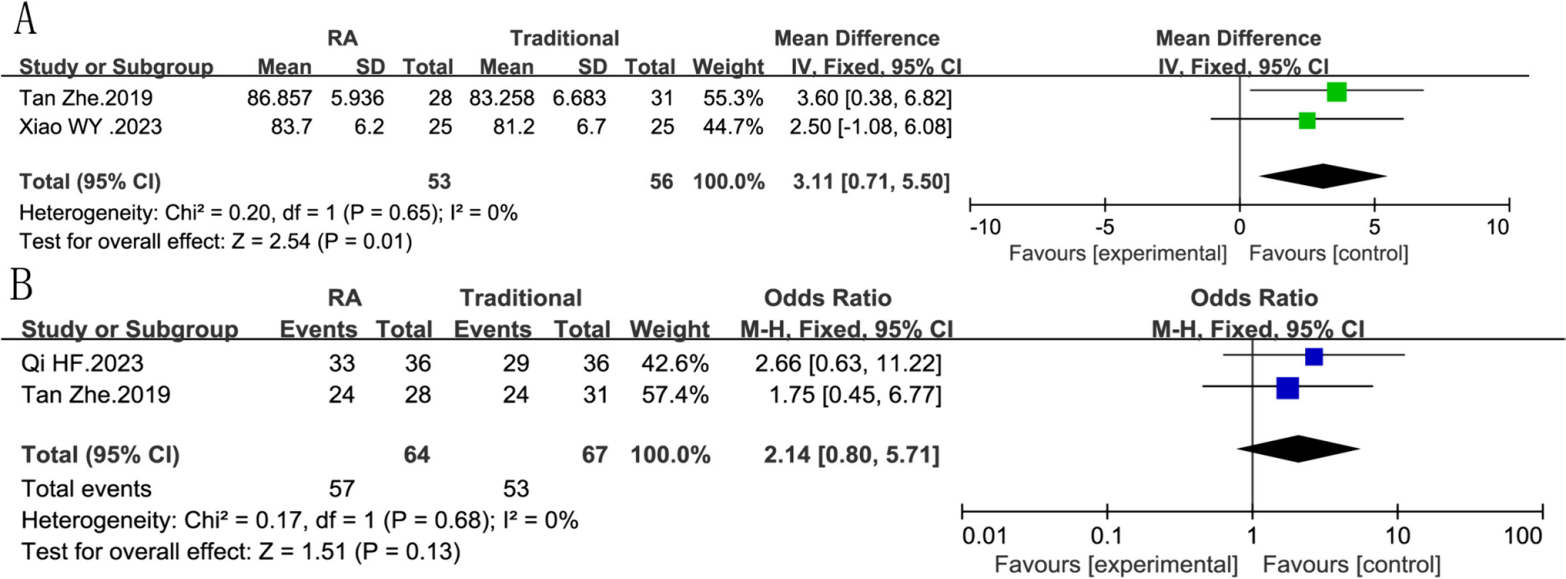
Forest plot diagram showing the Harris score and excellent and good rate **A** Harris score of robot-assisted vs traditional fixation, **B** excellent and good rate of robot-assisted vs traditional fixation

Three studies with 181 patients supported the hip functional evaluation at 1 year. A fixed model was used in the Harris score and excellent and good rate, and no heterogeneity was appeared (Fig. 5A: Harris score, *I*^2^ = 0%, *P *= 0.65; Fig. 5B: excellent and good rate, *I*^2^ = 0%, *P *= 0.68). The Harris score evaluation of the robot-assisted group was significantly better, except the excellent and good rate. (Fig. 5A: Harris score, RR = 3.11, 95% CI 0.71, 5.50, *P *= 0.01; Fig. 5B: excellent and good rate, RR = 2.79, 95% CI 0.80, 5.71, *P *= 0.13).

#### Sensitivity analysis

**Fig. 6:**
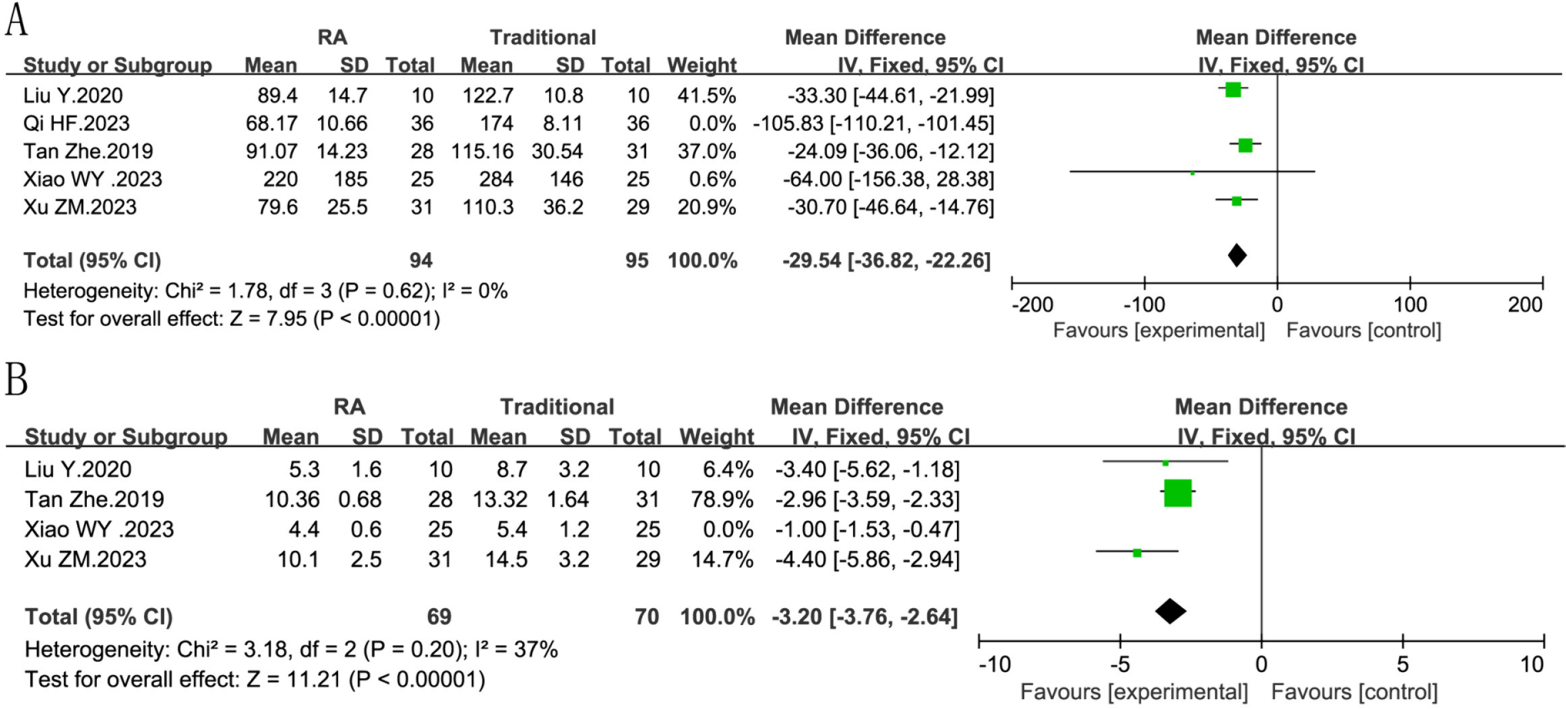
Forest plot diagram showing sensitivity analysis of intraoperative blood loss and intraoperative fluoroscopy times **A** intraoperative blood loss of robot-assisted fixation vs traditional fixation, **B** intraoperative fluoroscopy times of robot-assisted fixation vs traditional fixation

We performed sensitivity analyses for intraoperative blood loss (*I*^2^ = 0%, *P *= 0.62, Fig. 6A)and intraoperative fluoroscopy times (*I*^2^ = 37%, *P *= 0.20, Fig. 6B), the heterogeneity was reduced, and the superiority of robot-assisted was reflected.( intraoperative blood loss: RR = −29.54, 95% CI −36.82, −22.26 *P *< 0.00001,Fig. 6A; intraoperative fluoroscopy times: RR = −3.20, 95% CI −3.76, −2.64, *P *< 0.00001,Fig. 6B).

## Discussion

Intertrochanteric fracture is a common hip fracture, Proximal femoral nail antirotation(PFNA) is also the most common surgical treatment at present, its advantages are central fixation, fewer complications and good surgical results [17, 18]. With the development of various techniques and minimally invasive surgery, robot-assisted surgery has been paid more and more attention due to its advantages of small incision and high accuracy [19]. This article mainly analyzes the clinical effect of robot-assisted in the application of intertrochanteric fractures.

In our analysis, we found that robot-assisted therapy significantly shortened the procedure time, reduced intraoperative blood loss, and reduced the number of fluoroscopy times. These findings are consistent with the performance of current research on robot-assisted therapy in surgery and reflect the advantages of robots [20, 21]. In terms of surgery, XU’s article provides a further analysis of the operation time. When the overall time was divided into reduction time and operation time, robot-assisted performed well [13].

We conducted a sensitivity analysis of the heterogeneity of the two indicators. In the study of surgical bleeding, Qiu’s article was kicked out, and it was found that the heterogeneity disappeared. The main reason was that the bleeding volume between the two operations was too different, and the sample weight ratio was slightly higher, which affected the heterogeneity [12]. This article also puts forward the question of hidden bleeding, which needs further discussion on the difference of bleeding between the two surgeries, and whether it can be made up in the later stage according to the relevant hematological examination. In terms of fluoroscopy times, after excluding Xiao’s article, the heterogeneity also decreased significantly. Although in the original article, the data between the two were different according to statistical analysis, the average robot-assisted fluoroscopy was one-two times less, which was not obvious for the overall advantage [14].

In addition, in the one-time success rate of puncture and the good rate of screw placement, the robot showed highlight moments [14, 15]. In terms of puncture and TAD performance, the accuracy of the robot is reflected, which reduces the radiation exposure of doctors and patients, ensures that the screws are in a better position and the TAD value is better. It has advantages for young patients with stable reduction and early ambulation, but for elderly patients with serious conditions, the purpose of surgery is to solve pain and facilitate bed care, TAD value does not have to pursue perfection.

In terms of complications, DVT and death due to pulmonary embolism proposed in Xiao’s article are obviously related to bed stay, so minimally invasive surgery and early ambulation are more necessary [14, 22, 23]. In terms of functional scores, robot-assisted patients performed better on the Harris score. However, in the subjective evaluation of patients, there is no significant difference between the two, mainly because the Harris score includes pain, function, deformity and joint range of motion, which considers the range more comprehensive and more representative [24].

As the first meta-analysis on the use of robot-assisted in the treatment of intertrochanteric fractures, although the number of patients included was small, this will provide more reliable evidence for clinical practice.

## Conclusion

Based on the analysis of existing evidence, robot-assisted show an advantage over the traditional treatment in the operation time, intraoperative blood loss, intraoperative fluoroscopy times, pine insertion, TAD and hip function. In terms of excellent and good rate after operation did not show good aspect. The quality and quantity of experiments in this study is still relatively small. More large-scale, multicenter, high-quality studies are needed to confirm this result.

## Data Availability

The data that support the findings of this study are openly available in China National Knowledge Infrastructure and Pubmed at 1, 10.13795/j.cnki.sgkz.2023.09.019; 2, 10.2147/CIA.S412397; 3, 10.3969/j.issn.2095-4344.1290; 4, 10.3969/j.issn.2096-3351.2020.02.013; 5, 10.1111/os.13954.
